# Measuring overuse of continuous pulse oximetry in bronchiolitis and developing strategies for large-scale deimplementation: study protocol for a feasibility trial

**DOI:** 10.1186/s40814-019-0453-2

**Published:** 2019-05-15

**Authors:** Irit R. Rasooly, Rinad S. Beidas, Courtney Benjamin Wolk, Frances Barg, Christopher P. Landrigan, Amanda Schondelmeyer, Patrick W. Brady, Lisa M. McLeod, Christopher P. Bonafide, for the Pediatric Research in Inpatient Settings (PRIS) Network

**Affiliations:** 1 Section of Hospital Medicine, Children’s Hospital of Philadelphia, Buerger Center for Advanced Pediatric Care, 3500 Civic Center Blvd., 11th Floor, Philadelphia, PA USA; 2 Department of Psychiatry, Perelman School of Medicine, University of Pennsylvania, 3535 Market Street, Suite 3015, Philadelphia, PA USA; 3 Department of Medical Ethics and Health Policy, Perelman School of Medicine, University of Pennsylvania, Blockley Hall, 423 Guardian Drive, Philadelphia, PA USA; 4 Leonard Davis Institute of Health Economics, University of Pennsylvania, 3535 Market Street, Suite 3006, Philadelphia, PA USA; 5 Department of Family Medicine and Community Health, Perelman School of Medicine, University of Pennsylvania, Blockley Hall, 423 Guardian Drive, Philadelphia, PA USA; 6 Division of General Pediatrics, Department of Pediatrics, Boston Children’s Hospital, 300 Longwood Ave, Enders 1, Boston, MA USA; 7 Division of Sleep and Circadian Disorders, Departments of Medicine and Neurology, Brigham and Women’s Hospital, 75 Francis Street, Boston, MA USA; 8 Harvard Medical School, 25 Shattuck St., Boston, MA USA; 9 Department of Pediatrics, University of Cincinnati College of Medicine, 3333 Burnet Ave., MLC 9016, Cincinnati, OH USA; 10 Division of Hospital Medicine, Cincinnati Children’s Hospital Medical Center, 3333 Burnet Ave., MLC 9016, Cincinnati, OH USA; 11 James M. Anderson Center for Health Systems Excellence, Cincinnati Children’s Hospital Medical Center, 3333 Burnet Ave., MLC 7014, Cincinnati, OH USA; 12 Department of Pediatrics, Section of Hospital Medicine, Children’s Hospital Colorado, 13123 E. 16th Street, Aurora, CO USA; 13 Adult Child Consortium for Outcomes Research and Healthcare Delivery Sciences, University of Colorado School of Medicine, 13001 East 17th Place, Aurora, CO USA; 14 Center for Pediatric Clinical Effectiveness, Children’s Hospital of Philadelphia, Roberts Center for Pediatric Research, 2716 South Street, Philadelphia, PA USA

**Keywords:** Bronchiolitis, Overuse, Deimplementation, Implementation science, Pediatric hospital medicine

## Abstract

**Background:**

Deimplementation, the systematic elimination of low-value practices, has emerged as an important focus within implementation science. Bronchiolitis is the leading cause of infant hospitalization. Among stable inpatients with bronchiolitis who do not require supplemental oxygen, continuous pulse oximetry monitoring is recognized as an overused, low-value practice in pediatric hospital medicine. There is strong scientific evidence and practice guideline support for limiting pulse oximetry monitoring of stable children with bronchiolitis who do not require supplemental oxygen, yet the practice remains common. This study aims to (1) characterize the extent of this overuse in hospitals located in the USA and Canada, (2) identify barriers and facilitators of successful deimplementation of continuous pulse oximetry monitoring in bronchiolitis, and (3) develop consensus strategies for large-scale deimplementation. In addition to identifying feasible strategies for deimplementation, this study will test the feasibility of data collection approaches to be employed in a large-scale deimplementation trial.

**Methods:**

This multicenter study will be performed in approximately 38 hospitals in the Pediatric Research in Inpatient Settings Network. In Aim 1, we will determine the rate of overuse within each hospital by performing repeated cross-sectional observational sampling of continuous pulse oximetry monitoring of stable bronchiolitis patients age 8 weeks through 23 months who do not require supplemental oxygen. In Aim 2, we will use the Consolidated Framework for Implementation Research (CFIR) as a framework for semi-structured interviews with key stakeholders (physician, nurse, respiratory therapist, administrator, and parent) at the highest- and lowest-overuse hospitals to understand barriers and facilitators of continuous pulse oximetry monitoring deimplementation. In Aim 3, we will use a theory-based causal model to match the identified barriers and facilitators to potential strategies for deimplementation. Candidate strategies will be discussed with a panel of stakeholders from hospitals with high rates of overuse to assess feasibility and acceptability. A questionnaire ranking strategies based on feasibility, acceptability, and impact will be administered to a broader group of stakeholders to arrive at consensus about promising strategies for large-scale deimplementation to be tested in a subsequent trial.

**Discussion:**

Effective strategies for deimplementing continuous pulse oximetry monitoring of stable patients with bronchiolitis have not been well characterized. The findings of this study will provide further understanding of factors that facilitate deimplementation in pediatric hospital settings and provide pilot and feasibility data to inform a trial of large-scale deimplementation of this overused practice.

**Trial registration:**

Not applicable. This study does not meet the World Health Organization definition of a clinical trial.

**Electronic supplementary material:**

The online version of this article (10.1186/s40814-019-0453-2) contains supplementary material, which is available to authorized users.

## Background

Deimplementation, the systematic, structured reduction or elimination of low-value practices [1–3], has emerged as a distinct focus within the field of implementation science. Reduction of *overuse*, the provision of unnecessary services for which the potential for harm outweighs the potential for benefit [4, 5], is a key application of deimplementation. In pediatrics, research on overuse has been largely descriptive [6, 7]. While numerous candidate conditions for deimplementation have been identified [8–15], efforts to curb overuse have been limited. Application of rigorous implementation science methods to the development and evaluation of strategies for deimplementation is an essential next step to reducing pediatric overuse practices.

Continuous pulse oximetry (SpO_2_) monitoring in bronchiolitis is an important area of overuse in pediatric hospital medicine. Bronchiolitis is the leading cause of infant hospitalization with over 100,000 annual admissions at a cost of over $1.7 billion [7, 16]. Hospital treatment is primarily supportive [17], focused on optimizing hydration and treating hypoxemia, and often includes continuous SpO_2_ monitoring. Though there is strong evidence to suggest continuous SpO_2_ monitoring stable patients with bronchiolitis is unnecessary and may even pose risk, the practice remains widespread. While formal study of the barriers to deimplementing this practice have not yet been undertaken, we speculate that potential reasons for the practice continuing include perceptions of a higher degree of safety conferred when patients are monitored, entrenched practices and customs to monitor patients, and clinicians’ perceptions that families prefer that their children be monitored.

Outpatient studies of infants with bronchiolitis well enough to be discharged home from the emergency department without admission demonstrate that transient hypoxemia associated with bronchiolitis is common and likely of little clinical consequence [18]. Conversely, continuous monitoring has been associated with increased rates of admission, longer length of stay, more treatment with supplemental oxygen, and higher hospital cost, without improving outcomes [19, 20]. More generally, from a system level perspective, continuous SpO_2_ monitoring overuse incurs the potential hazards of *alarm fatigue*. Alarm fatigue is a well-described phenomenon in which exposure to high rates of false alarms results in desensitization, leading to ignored or slowed responses to all alarms [21–24].

The safety of reducing/eliminating continuous pulse oximetry of stable patients with bronchiolitis has been demonstrated in quality improvement initiatives [25] and a randomized controlled trial [19]. Both the American Academy of Pediatrics [17] and the Society of Hospital Medicine Choosing Wisely in Pediatric Hospital Medicine workgroup [8] support limiting monitoring of children with bronchiolitis. The American Academy of Pediatrics Bronchiolitis Clinical Practice Guideline states that clinicians may choose not to use continuous SpO_2_ monitoring for children with bronchiolitis [17]. Choosing Wisely Guidelines state more firmly that clinicians should not use continuous SpO_2_ monitoring in children with acute respiratory illness unless they are receiving supplemental oxygen [8].

Despite the guidelines, the practice of using continuous SpO_2_ monitoring in stable bronchiolitis patients of supplemental oxygen remains common. A community hospital-based quality improvement collaborative reported post-intervention rates of continuous monitoring approaching 50%, with marked variability between hospitals [26]. In order to develop a successful and broadly accepted intervention to reduce this potentially harmful practice, it is important to fully understand the barriers, facilitators, and contextual factors influencing (and impeding) successful deimplementation.

### Specific aims

The specific aims of this study are:

1. To measure baseline SpO_2_ monitoring overuse rates in children with bronchiolitis not requiring supplemental oxygen in 38 Pediatric Research in Inpatient Settings Network hospitals.
2. To identify barriers and facilitators to deimplementing SpO_2_ monitoring in children with bronchiolitis by conducting semi-structured interviews with key stakeholders at the highest- and lowest-overuse hospitals.
3. Informed by contextual analysis, develop a multifaceted deimplementation strategy for SpO_2_ monitoring overuse in children with bronchiolitis that will serve as the intervention to be tested in a subsequent clinical trial.

This study will be one of the first comprehensive evaluations of an overused practice in pediatric hospital medicine, providing insight into the state of overuse, the variation in overuse across hospitals, and the factors that contribute to overuse in a wide variety of hospital settings. Applying rigorous and reproducible methods to the study of deimplementation practices and obtaining a deep, contextual understanding of barriers and facilitators to deimplementation are essential for identifying strategies capable of producing large-scale, sustained reductions in overuse of this widespread practice. Specifically, contextual analysis will be used in the following ways: (1) in Aim 2, the organizational and social contexts relevant to monitoring overuse and its deimplementation will be queried and analyzed along with the barriers and facilitators that we seek to identify, and (2) in Aim 3, we will build upon the knowledge of organizational and social contexts in order to design a strategy or menu of strategies that can be tailored, if appropriate, to different organizational and social contexts.

Understanding why current pulse oximetry monitoring recommendations are (or are not) adhered to is essential to developing deimplementation strategies that will result in broad, sustained uptake. Findings of this study, including the feasibility of our approach to data collection, will be used to inform development of a hybrid effectiveness-implementation trial [27] of continuous SpO_2_ monitoring deimplementation: the Eliminating Monitor Overuse: pulse oximetry (EMO: SpO_2_) trial. Moreover, using a systematic approach to the study of deimplementation will allow for identification of strategies that are broadly generalizable to efforts to reduce overuse in pediatric hospital settings across a wide range of health conditions.

## Methods

### Study setting

This study will be performed within the Pediatric Research in Inpatient Settings Network (PRIS). PRIS is an independent, hospital-based research network that aims to improve the health of and healthcare delivery to hospitalized children and their families. The 117 member-hospitals range in geographic location, size, staffing models, and care practices. Hospitals are categorized as (1) freestanding children’s hospitals (standalone hospitals devoted entirely to the care of children including a full range of pediatric subspecialty services), (b) children’s hospitals within hospitals (general medical centers that care mainly for adult patients and include a pediatric department offering a full range of pediatric subspecialty services), and (c) community hospitals (general medical centers that care mainly for adult patients and include a pediatric department offering limited or no pediatric subspecialty services).

### Aim 1: measure baseline SpO_2_ monitoring overuse in bronchiolitis

We will use rigorous observational methods to test the hypothesis that SpO_2_ overuse exceeds 30% averaged across sites and that there is sufficiently wide variation in overuse rates between sites to distinguish high and low overuse hospitals. This hypothesis is based on quality improvement initiatives focused more generally at reducing a range of overused practices in bronchiolitis [26] and internal estimates from participating hospitals. Our study builds on prior work by evaluating SpO_2_ monitoring at a diverse range of hospitals and employing a standardized approach to arrive at accurate, generalizable, and reproducible estimates of practice prevalence.

We will perform repeated cross-sectional observational sampling of SpO_2_ monitor use among 60 children at each site who are hospitalized during peak bronchiolitis season (December 2018 through March 2019). Eligible patients include children 8 weeks through 23 months old with a primary diagnosis of bronchiolitis in their most recent physician progress note, who are not requiring supplemental oxygen or “room air flow” of 21% oxygen administered via nasal cannula at any flow rate while hospitalized on non-ICU wards. Patients will be excluded if any of the following criteria are present: < 28 weeks completed gestation, cyanotic congenital heart disease, pulmonary hypertension, home oxygen requirement, neuromuscular disease, immunodeficiency, and cancer.

Data collection will be operationalized at sites during periodic daytime (between 10 AM and 4 PM) and nighttime (between 11 PM and 7 AM) in-person observational “rounds.” Investigators will aim to collect 50% of their total number of observations during daytime rounds and 50% during nighttime rounds. During a data collection round, site investigators will identify all patients currently admitted to non-ICU pediatric wards with bronchiolitis by reviewing the census of each unit that cares for children with bronchiolitis and examining the charts of patients meeting age criteria to determine eligibility. They will then walk to the bedside of each eligible infant with bronchiolitis to confirm that supplemental oxygen and room air flow are off and determine the current continuous monitoring status (SpO_2_, electrocardiographic, neither, both). Site investigators will transmit data using REDCap, a secure research data management tool [28]. A draft data collection form is provided (Additional file 1).

The primary outcome is the overall rate of SpO_2_ monitoring overuse of patients with bronchiolitis within each hospital, defined as the number of continuously SpO_2_-monitored patients with bronchiolitis who are not requiring supplemental oxygen or room air flow divided by the total number of patients with bronchiolitis who are not requiring supplemental oxygen or room air flow. This study will also measure the following secondary outcomes: (1) use of intermittent SpO_2_ measurement, (2) use of continuous electrocardiographic monitoring (to explore potential co-occurrence of monitor overuse), and (3) the association between orders and actual monitoring. Overuse rate rankings by hospital type will be generated. We will explore differences in overuse between day and night, race/ethnicity, hospital type, and co-occurrence of electrocardiographic monitoring overuse.

### Feasibility

In terms of statistical power, engaging 38 sites (allowing for ~ 20% attrition) will enable us to achieve an acceptable range of low and high overuse hospitals within each hospital type. We determined sample size based on the width of the 95% confidence interval of the point estimate of the site-level overuse rate. The intent is to have a narrow enough width to separate the lowest overuse hospitals (projected 0–30% overuse) from the highest overuse hospitals (projected 70–90% overuse). We hypothesize that a total sample size of 60 patients per hospital will allow for an appropriate balance between data collection feasibility and precision of the point estimates (see Table 1) in this pilot study.

**Table 1:** 95% confidence intervals of overuse estimates across a range of within-hospital patient sample sizes and overuse rates

	*n* = 20	*n* = 60	*n* = 100
30% overuse	11.9–54.3%	18.8–43.2%	21.2–40.0%
70% overuse	45.7–88.1%	56.8–81.2%	60.0–78.8%

Feasibility of our site recruitment and Aim 1 data collection approaches are important outcomes of this trial and will inform the final design of the EMO SpO_2_ trial, a large scale, cluster randomized type 3 hybrid effectiveness-implementation trial [27]. In terms of recruitment and samples size, we have performed a preliminary power calculation for the subsequent EMO SpO_2_ trial using established methods for cluster-randomized trials [29]; however, the ultimate sample size of the EMO SpO_2_ trial will be derived based on findings of this pilot. The thresholds for evaluating feasibility of the recruitment and engagement strategies of this pilot will be defined as (1) the number of participating PRIS sites that complete 60 observations will be 24 or greater (based on a target of 18 as specified in the preliminary power calculation allowing for 25% attrition), and (2) the number of participating PRIS sites that complete at least 40% of observations overnight will be 24 or greater. Not attaining these thresholds will trigger design changes to the EMO SpO_2_ trial.

Evaluating the association between monitoring orders and observationally obtained data about monitoring, one of the secondary outcomes of this Aim, is important in establishing the validity and feasibility of the data collection protocols for the EMO SpO_2_ trial. This pilot will employ a personnel-intensive observational approach to determine which patients are continuously monitored. We hypothesize that this approach is feasible and will be more accurate than examining orders to determine true continuous monitoring status across a wide range of hospitals with varying chart documentation and physiologic monitor data infrastructures. We will determine sensitivity, specificity, and positive and negative predictive values for the relationship between the presence of an order for continuous SpO_2_ monitoring and actual monitoring status to inform monitoring in the EMO SpO_2_ trial, overall and within individual hospitals. If the presence of a monitoring order exceeds 85% for sensitivity and specificity, either overall or in specific sites, we will consider using the order as an accurate indicator for monitoring in the subsequent trial.

### Aim 2: identify barriers and facilitators to deimplementing SpO_2_ monitoring in bronchiolitis

We will perform semi-structured qualitative interviews with key stakeholders at hospitals with the highest and lowest rates of SpO_2_ overuse to explore clinical, political, interpersonal, and normative factors surrounding pulse oximetry overuse in bronchiolitis. Interviews will also elucidate processes of initiating and discontinuing pulse oximetry. Then, using the Consolidated Framework for Implementation Research (CFIR) as a guiding framework [30], we will identify barriers and facilitators of deimplementing continuous SpO_2_ monitoring of stable infants who are breathing room air. CFIR is a widely accepted, pragmatic meta-theoretical framework of constructs that influence effective implementation including *Intervention Characteristics* (for instance, perceptions of the evidence supporting continuous SpO_2_ monitoring), the *Outer Setting* (such as parental preferences or institutional policies and protocols), the *Inner Setting* (for example, unit/department cultural norms related to monitoring), and *Characteristics of Individuals* (including knowledge of the clinical guidelines or beliefs about risks of harm from brief desaturations). Finally, using an approach derived from the Intervention Mapping methodology [31], we will ask participants to identify key factors and behaviors associated with adoption, implementation, and sustaining intermittent SpO_2_ measurement instead of using continuous SpO_2_ monitoring. A draft interview guide is provided (Additional file 2).

With respect to sample size, we will use a purposive sampling strategy to select a sample of an anticipated 75 stakeholders from the two highest overuse and the two lowest overuse hospitals within each hospital type (freestanding children’s hospitals, children’s hospitals within general hospitals, and community hospitals). We plan to conduct approximately 15 interviews with representatives from each stakeholder group (physicians, nurses, respiratory therapists, administrators, parents) until thematic saturation is achieved within each group. If we fail to achieve thematic saturation after 15 interviews, we will make adjustments to the study budget to allow us to perform additional interviews until thematic saturation is achieved. Parent interviews will focus on monitoring preferences [32].

### Qualitative analysis using the CFIR implementation framework

Data management and analysis of interview transcripts will be supported by the use of NVivo software. Both a priori and grounded theory methods [33] will be used in the coding process. The CFIR framework constructs will serve as an initial coding structure (a priori aspect), but we will also allow new concepts to emerge and become part of the coding scheme (inductive aspect). Process maps [34, 35] delineating SpO_2_ monitoring initiation and discontinuation processes will be created for each hospital based on descriptions of the processes gleaned from the qualitative interviews. Process maps will specifically identify process failures (barriers) that lead to unnecessary monitoring and successful processes (facilitators) that help clinicians avoid unnecessary monitoring.

Initially, team members will complete coding and process maps independently on a common subset of interviews and compare results to assess the reliability and robustness of the coding scheme. Disagreements will be resolved through team discussion. Prior to proceeding with the remainder of the analysis, we will repeat independent coding and measure inter-rater reliability using Cohen’s kappa. If an “almost perfect” kappa of 0.81 or higher [36] is achieved, we will largely employ single reviewer coding, with dual coding for 20% of transcripts. If the kappa is below 0.81, we will dual code all transcripts. Emerging codes, coding challenges, and coding disagreements will be discussed during standing team meetings.

Coding results will result in the identification of key factors that affect the ultimate outcome. These factors will be incorporated into a Qualitative Comparative Analysis (QCA), a mixed methods approach to examine complex combinations of explanatory factors [37]. This analytic approach is useful for identifying “causal complexity,” for instance, situations in which factors may only impact outcomes in the presence of other factors [37, 38]. In QCA, factors identified as salient will be used to create a “truth table” in which each hospital is a row and each identified factor is a column [39, 40]. Cells will indicate the presence or degree of presence of each factor at each hospital, allowing for identification of combinations of factors that appear necessary to achieve the targeted deimplementation outcomes.

### Aim 3: development of a multifaceted deimplementation strategy

Using the CFIR constructs and elements of the Theory of Planned Behavior (TPB) [41], barriers and facilitators identified in our qualitative analysis will be categorized and matched with potential strategies for deimplementation. Potential strategies will be drawn from our stakeholder interviews, Expert Recommendations for Implementing Change (ERIC) strategies [42], the broader implementation science literature on behavior change, and the literature on deimplementation of electrocardiographic monitoring in hospitalized adults [43–50]. Leveraging these implementation science and behavioral science theories and recommendations (CFIR, Theory of Planned Behavior, and ERIC strategies), we will construct a table that serves as a “crosswalk” between the identified barriers or facilitators and potential deimplementation strategies (e.g., Table 2).

**Table 2:** Examples of matching barriers that may be identified in Aim 2 to deimplementation strategies

Examples of potential barriers to deimplementation	→	Consolidated Framework for Implementation Research constructs [30]	→	Elements of adapted Theory of Planned Behavior [51]	→	Expert Recommendations for Implementing Change strategy types [42]
Belief that continuous monitoring is a safety net	→	Characteristics of individuals: knowledge and beliefs	→	Behavioral beliefs contributing to attitudes	→	Educational outreach visits, build a coalition, learning collaborative
Reluctance to change comfortably entrenched practice of continuously monitoring all infants	→	Characteristics of individuals: self-efficacy, Inner setting: implementation climate	→	Self-efficacy, organizational culture and climate, habit	→	Provide reminders (clinical decision support), audit and feedback, clinical champions, learning collaborative
Monitoring because of perceived parental preference that infant be monitored (preference may be real or assumed)	→	Characteristics of individuals: knowledge and beliefs, Outer setting: patient needs	→	Behavioral beliefs contributing to attitudes, self-efficacy	→	Educational outreach visits, clinical champions, involving parents as active participants in deimplementation effort

Potential strategies (adapted to specifically address deimplementation of continuous SpO_2_ monitoring overuse) with supporting evidence-based literature will be presented to a Strategy Development Panel focus group and refined based on panel input. The panel will be comprised of 12 of the interviewed stakeholders from high overuse hospitals including at least two physicians, nurses, respiratory therapists, administrators, and parents. After presenting each potential strategy and the barriers/facilitators it is meant to address, we will use open-ended questions to inquire about the feasibility, acceptability, and anticipated impact of each potential strategy. For example, if a potential strategy involves building clinical decision support alerts into the electronic health record, we will evaluate feasibility by inquiring about the information technology capacity to develop new electronic health record alerts; the timeline for development; and their history of successfully developing, implementing, and evaluating alerts for hospitalized children. The facilitator will apply selected methods from nominal group technique [52] in order to ensure that all members of the panel are able to meaningfully contribute. This will include (1) a group sample size of approximately 12 panelists; (2) allowing a period of time after the potential strategy is introduced for each panelist to write down their initial impressions and ideas; (3) polling each group member, one at a time, until every idea is recorded; and (4) permitting time for discussion, clarification, and modification of each idea.

Detailed specifications [53] of the ten highest impact strategies, as determined by the Strategy Development Panel, will be distributed to all stakeholders previously interviewed from high overuse hospitals (*n* = 30) and site investigators from ten additional hospitals that had overuse rates greater than or equal to the median overuse rate. Using a questionnaire, we will measure stakeholder intentions, attitudes, perceived norms, and self-efficacy/perceived control with respect to each potential strategy and ask stakeholders to rate each potential strategy based on anticipated feasibility, acceptability, and impact. Survey results will be reviewed by the study team and advisory committee, the Strategy Development Panel, and the National Heart, Lung, and Blood Institute Project Scientists to arrive at consensus about the most promising individual strategies to include in a multi-component deimplementation strategy.

## Discussion

This protocol outlines the design of a study that will bridge the gap between the guidelines and evidence that support deimplementation and the actual process of deimplementing continuous SpO_2_ monitoring in stable infants and children with bronchiolitis who do not require supplemental oxygen.

Given the prevalence of this practice in spite of scientific and professional consensus about its lack of utility, there is a critical need for systematic evaluation of the barriers and facilitators to deimplementation in order to design more effective approaches to deimplementation. Sustained uptake of EMO SpO_2_ trial protocols for deimplementation hinges on careful elucidation of the current factors influencing adoption of the evidence practice and work with stakeholders to design specific and targeted strategies that address the issues identified. Only with an understanding of the contextual factors contributing to the current practice state will we be able to identify deimplementation strategies with broad feasibility, acceptability, and effectiveness.

In their review, “Letting Go: Conceptualizing intervention de-implementation in public health and social service settings,” McKay et al. describe the process of deimplementation as consisting of identifying targets for deimplementation, assessing the context in which deimplementation is to occur, putting into place strategies to support deimplementation practices, and evaluating outcomes of deimplementation efforts [54]. The second step, contextual analysis of barriers and facilitators to a specific deimplementation practice, is a crucial but often overlooked element of successful deimplementation [54]. By focusing on this aspect of deimplementation, our protocol will serve to not only increase the likelihood of successful deimplementation of this specific practice (continuous SpO_2_ monitoring in stable children with bronchiolitis) but will also add generalizable knowledge about how to conduct systematic contextual analysis as it relates to deimplementation and enhance our understanding of the factors contributing to successful deimplementation.

There are a few potential threats and limitations to successful execution of this study. One potential threat is lower or more homogeneous than anticipated rates of SpO_2_ overuse. Though this seems unlikely given internal estimates and recent community hospital data, we could consider unit-based (as opposed to hospital based) analysis or apply a historic focus to qualitative analysis, evaluating prior barriers, facilitators, and best practices that led to successful deimplementation. Another potential limitation would be if the Strategy Development Panel determines that deimplementation strategies should be tailored for individual hospitals or hospital types; we will present promising strategies as a “menu” of potential options rather than as a unified, uniform approach.

This multi-site study will produce accurate rates of continuous SpO_2_ monitoring overuse among stable infants with bronchiolitis; detailed analysis of factors that perpetuate and disrupt overuse; and consensus-derived, theory driven strategies for addressing this prevalent clinical issue. Strengths of this approach include our diverse sample of hospital and stakeholders, which will allow for rich contextual analysis, and the application of multiple implementation science frameworks to the nascent field of deimplementation. As the field of pediatric hospital medicine evolves, it is crucial to develop sound methodologies for implementation and deimplementation to facilitate consistent delivery of high-quality inpatient care.

## Additional files

Additional file 1: This is a draft of the data collection form proposed for use in Specific Aim 1. (PDF 44 kb)

Additional file 2: This is a draft of the interview guide proposed for use when interviewing physicians from high overuse hospitals in Specific Aim 2. (PDF 142 kb)

## Abbreviations

AAP: American Academy of Pediatrics
CFIR: Consolidated Framework for Implementation Research
EMO: SpO_2_: Eliminating Monitor Overuse: pulse oximetry
ERIC: Expert Recommendations for Implementing Change
PRIS: Pediatric Research in Inpatient Settings Network
QCA: Qualitative Comparative Analysis
TPB: Theory of Planned Behavior
