# Supplementary material for: Measuring overuse of continuous pulse oximetry in bronchiolitis and developing strategies for large-scale deimplementation: study protocol for a feasibility trial
Source: Pilot Feasibility Stud. 2019 May 15;5:68. doi: 10.1186/s40814-019-0453-2 (PMC6518681; doi:10.1186/s40814-019-0453-2)
Supplement: Supplementary file 1 — This is a draft of the data collection form proposed for use in Specific Aim 1. (PDF 44 kb) [file 40814_2019_453_MOESM1_ESM.pdf]

pulse oximetry overuse assessment

Please complete the questions below.

Thank you!

Instructions

Complete one of these forms for each patient
INCLUSION PROCESS: First, screen the charts of all hospitalized patients age 8 weeks through 23 months old on all non-ICU units that care for patients with bronchiolitis to determine if they have a primary diagnosis of bronchiolitis based on physician progress notes
EXCLUSION BASED ON CHART SCREENING: Of the patients who meet inclusion criteria of having a primary diagnosis of bronchiolitis, exclude patients with any of these conditions:
extreme prematurity (< 28 weeks) cyanotic congenital heart disease pulmonary hypertension home oxygen or positive pressure ventilation requirement tracheostomy neuromuscular disease immunodeficiency cancer
EXCLUSION BASED ON WALKING TO THE BEDSIDE: For patients who meet the initial inclusion and exclusion criteria above, walk to each of their bedsides to determine if they are receiving supplemental oxygen. If they are receiving any supplemental oxygen or any nasal cannula flow (even if it's 21% FiO2), they are excluded, so do not collect any data on those patients. Only collect data on patients who are not receiving any supplemental oxygen or flow based on your visual confirmation in the room.
For patients who are not receiving any supplemental oxygen or flow based on your visual confirmation in the room, proceed with the rest of data collection.

Chart screening for inclusion/exclusion

Patient age:	◯ 8 weeks through 5 months<br>◯ 6 months through 11 months<br>◯ 12 months through 17 months<br>◯ 18 months through 23 months<br>◯ Other (patient ineligible, STOP and cancel this entry. Do not enter this patient's data into REDCap)
Does the patient have a primary diagnosis of bronchiolitis based on the most recent physician note?	◯ No or uncertain (patient ineligible, STOP and cancel this entry. Do not enter this patient's data into REDCap)<br>◯ Yes - proceed with data collection
Does the patient have any of these conditions? extreme prematurity (< 28 weeks) cyanotic congenital heart disease pulmonary hypertension home oxygen or positive pressure ventilation requirement tracheostomy neuromuscular disease immunodeficiency cancer	◯ No - proceed with data collection<br>◯ Yes (patient ineligible, STOP and cancel this entry. Do not enter this patient's data into REDCap)
Is a numeric gestational age listed in this patient's chart?	◯ No<br>◯ Yes
Patient gestational age:	◯ 28 0/7 to 33 6/7 weeks<br>◯ 34 0/7 to 36 6/7 weeks<br>◯ 37 0/7 weeks and above
If no numeric gestational age is listed, is the patient described as "full term" in the chart?	◯ No<br>◯ Yes

If no numeric gestational age is listed, is the patient described as "premature" or "preterm" in the chart?	◯ No<br>◯ Yes (patient ineligible, STOP and cancel this entry. Do not enter this patient's data into REDCap)

Obtain this data by walking to the patient's bedside and documenting your observations

Based on observing them at the bedside, is the patient currently receiving any supplemental oxygen (FiO2 >21%)?	◯ No (continue data collection)<br>◯ Yes (patient ineligible, STOP and cancel this entry. Do not enter this patient's data into REDCap)
Based on observing them at the bedside, is the patient currently receiving any nasal cannula flow at an FiO2 of 21% (room air flow)?	◯ No (continue data collection)<br>◯ Yes (patient ineligible, STOP and cancel this entry. Do not enter this patient's data into REDCap)
Based on observing them at the bedside, is the patient being continuously pulse oximetry monitored right now?	◯ No<br>◯ Yes
Based on observing them at the bedside, is the patient being continuously cardiorespiratory monitored using chest leads to measure heart rate and/or respiratory rate right now?	◯ No<br>◯ Yes

Obtain this data from the patient's chart

According to the chart, was the patient ever on supplemental oxygen or room air flow previously during this hospital visit, including in the emergency department?	◯ No<br>◯ Yes<br>◯ Unknown
According to the chart, how long has the patient been off supplemental oxygen and flow?	◯ Off supplemental oxygen and flow less than 1 hour<br>◯ Off supplemental oxygen and flow 1 to less than 2 hours<br>◯ Off supplemental oxygen and flow 2 to less than 3 hours<br>◯ Off supplemental oxygen and flow 4 to less than 6 hours<br>◯ Off supplemental oxygen and flow 6 to less than 12 hours<br>◯ Off supplemental oxygen and flow 12 to less than 24 hours<br>◯ Off supplemental oxygen and flow 24 hours or more<br>◯ Cannot be determined from chart

Does the patient have an active CONTINUOUS pulse oximetry monitoring order in the chart right now?	◯ N/A: we don't place orders for continuous pulse oximetry monitoring at our hospital<br>◯ No<br>◯ Yes, and it says this patient should be continuously monitored all the time<br>◯ Yes, and it says the patient should only be monitored when specific conditions are met (such as when asleep), and the patient currently MEETS those conditions (so should be monitored now)<br>◯ Yes, but it says the patient should only be monitored when specific conditions are met (such as when asleep), and the patient currently DOES NOT MEET those conditions (so should not be monitored now)
Does the patient have an active CONTINUOUS cardiorespiratory monitoring order (to use chest leads to measure heart rate and/or respiratory rate) in the chart right now?	◯ N/A: we don't place orders for continuous cardiorespiratory monitoring at our hospital<br>◯ No<br>◯ Yes, and it says this patient should be continuously monitored all the time<br>◯ Yes, and it says the patient should only be monitored when specific conditions are met (such as when asleep), and the patient currently MEETS those conditions (so should be monitored now)<br>◯ Yes, but it says the patient should only be monitored when specific conditions are met (such as when asleep), and the patient currently DOES NOT MEET those conditions (so should not be monitored now)
Patient sex:	◯ Male<br>◯ Female<br>◯ Not specified
Patient ethnicity according to NIH definitions:	◯ Hispanic or Latino<br>◯ Not Hispanic or Latino<br>◯ Other<br>◯ Unknown
Patient race according to NIH definitions:	◯ White<br>◯ Black or African American<br>◯ Asian<br>◯ American Indian or Alaska Native<br>◯ Native Hawaiian or Other Pacific Islander<br>◯ More than one race<br>◯ Other<br>◯ Unknown
Does the patient have any of these devices? (these are not exclusions)	☐ Short-term feeding tube (e.g. nasogastric)<br>☐ Long-term feeding tube (e.g. gastrostomy)<br>☐ Central venous line<br>☐ None of the above
Does the patient have any of these historical characteristics? (these are not exclusions)	☐ History of apnea during this illness (at home or in hospital)<br>☐ History of cyanosis during this illness (at home or in hospital)<br>☐ History of ICU stay earlier in this admission<br>☐ None of the above

Does the patient have any of these forms of chronic neurologic impairment?<br>(these are not exclusions)	☐ Static encephalopathy<br>☐ Cerebral palsy<br>☐ Hydrocephalus<br>☐ Spina bifida<br>☐ Epilepsy/seizure disorder<br>☐ Hypotonia<br>☐ None of the above

Data collection rounds info

Name of the IRB approved study team member entering this data:	__________________________________<br>(Your name)
Hospital name:	__________________________________
Name of unit this patient is hospitalized on:	__________________________________
Date data collected:	__________________________________
Time data collected:	○ Day (10AM - 4PM)<br>○ Night (1AM - 7AM)
