# Supplementary material for: Measuring overuse of continuous pulse oximetry in bronchiolitis and developing strategies for large-scale deimplementation: study protocol for a feasibility trial
Source: Pilot Feasibility Stud. 2019 May 15;5:68. doi: 10.1186/s40814-019-0453-2 (PMC6518681; doi:10.1186/s40814-019-0453-2)
Supplement: Supplementary file 2 — This is a draft of the interview guide proposed for use when interviewing physicians from high overuse hospitals in Specific Aim 2. (PDF 142 kb) [file 40814_2019_453_MOESM2_ESM.pdf]

Specific Aim 2

Draft interview guide for physicians from high overuse hospitals

Introduction

Today I'd like to discuss the role of pulse oximetry monitoring in patients with bronchiolitis with you. Our research team is in the process of developing an intervention aimed at promoting the use of *intermittent* pulse oximetry measurement instead of *continuous* pulse oximetry monitoring in patients with bronchiolitis who are stable on room air, not requiring any supplemental oxygen. When I use the term "intervention" during our interview, that is what I am referring to.

The goal of today's interview is to discuss how clinicians at your hospital approach pulse oximetry use in bronchiolitis. We will discuss barriers and facilitators to reducing the use of continuous pulse oximetry at your hospital in patients who are stable on room air. It's important for you to know that there are NO right or wrong answers. I'm most interested in your perspective.

Section 1: Understanding the process and exploring general barriers/facilitators

Q (Role of oximetry): First, I'd like to hear your general thoughts about the role that continuous monitoring plays in caring for infants with bronchiolitis at your hospital. This includes pulse oximetry monitoring as well as monitoring heart rhythm, heart rate, and respiratory rate with electrocardiographic leads.

Q (Reaction to data): This past winter, we measured how often your hospital used continuous pulse oximetry in patients with bronchiolitis when they were not requiring supplemental oxygen. We found that the overuse rate was ___%, meaning that ___% of patients age 0-23 months who had a diagnosis of bronchiolitis and were breathing room air were being continuously monitored with pulse oximetry. Can you tell me if that percentage seems to make sense based on what you experience in your role at the hospital, or if it is surprising, and why?

Q (Prior experience): Have you or your hospital had any prior experience implementing interventions aimed at reducing unnecessary pulse oximetry monitoring in bronchiolitis? If so, can you tell me about them, and how they went?

Q (Initiation process): Tell me about the process of <u>initiating</u> continuous pulse oximetry in bronchiolitis patients at your hospital. I'm interested in the steps that need to occur to start a patient on continuous monitoring, and who is responsible for each step. I'm interested in all roles, including physicians, nurses, technicians, respiratory therapists, et cetera.

- *Explore each step for detailed descriptions of the who, what, where, when, why, and how. We will be generating a process map for this, so detail is important. Identify any areas where there is diffusion of responsibility between roles.*

Q (Discontinuation process): Tell me about the process of <u>discontinuing</u> continuous pulse oximetry in bronchiolitis patients at your hospital. I'm interested in the steps that need to occur to stop continuously monitoring a patient, and who is responsible for each step. I'm interested in all roles, including physicians, nurses, technicians, respiratory therapists, et cetera.

- *Explore each step for detailed descriptions of the who, what, where, when, why, and how. Identify any areas where there is diffusion of responsibility between roles.*
- **Probe:** Is there an amount of time between when a patient's supplementary oxygen is stopped and when continuous pulse oximetry monitoring is discontinued?

Q (Process failures): Can you give me some examples of when the above processes don't work well, and patients end up being monitored unnecessarily even though they are not requiring supplemental oxygen?

Q (General barriers): I'd like to hear your thoughts about any barriers you have experienced to intermittently measuring the pulse oximetry value instead of using continuous pulse oximetry monitoring in bronchiolitis patients who are not requiring supplemental oxygen. By barriers, I mean anything that has gotten in the way of launching, implementing or sustaining this practice in your setting.

Q (General facilitators): Next, I'd like to hear your thoughts about any facilitators you have experienced to intermittently measuring the pulse oximetry value instead of continuous pulse oximetry monitoring in bronchiolitis patients who are not requiring supplemental oxygen. By facilitators, I mean anything that has made it easier to launch, implement, or maintain this practice in your setting.

Section 2: Consolidated Framework for Implementation Research informed questions

I. Intervention Characteristics

Q (Evidence strength): What do you think of the evidence behind intermittently measuring the pulse oximetry value instead of using continuous pulse oximetry monitoring in bronchiolitis patients who are not requiring supplemental oxygen?

Q (Evidence strength): What do your co-workers think about the evidence behind intermittently measuring the pulse oximetry value instead of using continuous pulse oximetry monitoring in bronchiolitis patients who are not requiring supplemental oxygen?

Q (Evidence strength): What does your leadership think about the evidence behind intermittently measuring the pulse oximetry value instead of using continuous pulse oximetry monitoring in bronchiolitis patients who are not requiring supplemental oxygen?

Q (Complexity): How complicated would it be to change practice so that bronchiolitis patients who are not requiring supplemental oxygen have their pulse oximetry value measured intermittently instead of being continuously monitored?

II. Outer Setting

Q (Patient needs & resources): How do you anticipate that parents of bronchiolitis patients served by your hospital will respond to a practice shift of using less continuous pulse oximetry monitoring?

- **Probe:** Will they even notice? Do parents ever express their preferences or expectations about continuous monitoring? If so, what are the reasons they share for having those preferences or expectations? Do any of them report using pulse oximetry-based baby monitors at home?

Q (Peer pressure): Can you tell me about any other organizations that have shifted their practice to using less continuous pulse oximetry in bronchiolitis patients who are not requiring supplemental oxygen?

Q (Peer pressure): To what extent are other units within your organization shifting their practice to using less continuous pulse oximetry in bronchiolitis patients who are not requiring supplemental oxygen?

Q (External policies & incentives): What hospital or departmental policies would either help or hinder launching and sustaining an intervention to promote use of intermittent pulse oximetry measurement instead of using continuous pulse oximetry monitoring in bronchiolitis patients who are not requiring supplemental oxygen?

Q (External policies & incentives): What hospital protocols or care pathways for bronchiolitis would either help or hinder launching and sustaining an intervention to promote use of intermittent pulse oximetry measurement instead of using continuous pulse oximetry monitoring in bronchiolitis patients who are not requiring supplemental oxygen?

Q (External policies & incentives): Are there any other external pressures that would influence launching and sustaining an intervention to promote use of intermittent pulse oximetry measurement instead of using continuous pulse oximetry monitoring in bronchiolitis patients who are not requiring supplemental oxygen?

- **Probe:** For example, are there stories of adverse outcomes in patients who were unmonitored that seem to always come up when people discuss reducing the use of continuous monitoring? If so, who usually brings up those stories?

III. Inner Setting

Q (Structural characteristics): What kinds of infrastructure changes would be needed to accommodate an intervention to promote use of intermittent pulse oximetry measurement instead of using continuous pulse oximetry monitoring in bronchiolitis patients who are not requiring supplemental oxygen?

- **Probes:** Changes in scope of practice? Changes in policies? Changes in the electronic health record? What kind of approvals would be needed? Who would need to be involved?

Q (Culture): How would you describe the culture of your division or department with respect to continuous monitoring in bronchiolitis?

Q (Culture): How would you describe the culture of the unit or units where you most commonly work with respect to continuous monitoring in bronchiolitis, thinking about the interplay between physician, nursing, respiratory therapy, and administrative culture?

Q (Culture): How do you think the monitoring culture would make it easier or harder to implement an intervention to promote use of intermittent pulse oximetry measurement instead of using continuous pulse oximetry monitoring in bronchiolitis patients who are not requiring supplemental oxygen?

Q (Implementation climate): What do you expect would be the general level of receptivity in your organization to implementing an intervention to promote use of intermittent pulse oximetry measurement instead of using continuous pulse oximetry monitoring in bronchiolitis patients who are not requiring supplemental oxygen?

- **Probe:** Do you anticipate that units making improvements in reducing unnecessary pulse oximetry monitoring would be acknowledged or rewarded?
- **Probe:** Who in your organization would be most likely to resist this change, and why?

Q (Tension for change): Is there a perceived need to stop using continuous pulse oximetry monitoring in bronchiolitis patients who are stable on room air at your hospital? Why or why not?

Q (Available resources): What resources or support would be needed to effectively implement an intervention to promote use of intermittent pulse oximetry measurement instead of using continuous pulse oximetry monitoring in bronchiolitis patients who are not requiring supplemental oxygen at your hospital?

IV. Characteristics of Individuals

Q (Knowledge & beliefs): Do you think an intervention to promote use of intermittent pulse oximetry measurement instead of using continuous pulse oximetry monitoring in bronchiolitis patients who are not requiring supplemental oxygen would be effective at your hospital? Why or why not?

Q (Knowledge & beliefs): What is most uncomfortable to you about reducing the amount of continuous pulse oximetry monitoring that is occurring at your hospital?

Q (Knowledge & beliefs): How do you think reducing the amount of continuous pulse oximetry monitoring that is occurring at your hospital could improve patient outcomes?

- **Probe:** How do you balance your fears with these potential benefits?

Q (Self-efficacy): How confident are you that you will be able to successfully stop using continuous pulse oximetry monitoring in all bronchiolitis patients who are not requiring supplemental oxygen at your hospital?

- **Probe:** What else could be done to make that process easier?

Section 3: Intervention Mapping (Step 5) questions

In this final section, I will be asking you about the people who would be responsible for launching, implementing, and maintaining an intervention to promote use of intermittent pulse oximetry measurement instead of using continuous pulse oximetry in bronchiolitis patients.

Q (Role): Tell me about what role you'd have if this intervention were implemented in your setting.

- **Probe:** Do you anticipate that you would be involved in the decision to implement it, in actually implementing the intervention, and/or in helping ensure it is implemented correctly and consistently over time?

Q (Adopters): Who, specifically, in your health system would need to make the decision to use intermittent pulse oximetry measurement instead of using continuous pulse oximetry in bronchiolitis patients (e.g., hospital or department leadership, nursing department, chief quality officer)?

- **Probe:** What specific behaviors would indicate adoption of the practice?

Q (Implementers): Who, specifically, in your health system would actually be involved in the implementation of intermittent pulse oximetry measurement instead of continuous pulse oximetry in all bronchiolitis patients not requiring supplemental oxygen? Let's start by listing all of the potential people (doctors, nurses, medical assistants) who might be involved in the tasks necessary to avoid continuous monitoring and instead use intermittent oximetry measurement. Who would be best suited for which task? What training initiatives do you think would be necessary or helpful?

- *Explore how the practice might oversee quality of intervention delivery; communication between different clinicians; how this could be documented in the medical record.*

Q (Maintenance): Who would be responsible for ensuring that the intervention to promote use of intermittent pulse oximetry measurement instead of continuous pulse oximetry monitoring in bronchiolitis patients who are not requiring supplemental oxygen is implemented correctly and consistently over time? How would you know that the intervention has been maintained? In other words, what behavior would the key people responsible for maintenance engage in that would suggest the decision to sustain has been made (e.g., policies updated, clinical pathways, responsibilities clearly delineated)?

End

Thank you very much for taking part in this interview. We greatly appreciate the opportunity to learn from your perspectives.
